# Antisense transcripts of the expanded *C9ORF72* hexanucleotide repeat form nuclear RNA foci and undergo repeat-associated non-ATG translation in c9FTD/ALS

**DOI:** 10.1007/s00401-013-1192-8

**Published:** 2013-10-16

**Authors:** Tania F. Gendron, Kevin F. Bieniek, Yong-Jie Zhang, Karen Jansen-West, Peter E. A. Ash, Thomas Caulfield, Lillian Daughrity, Judith H. Dunmore, Monica Castanedes-Casey, Jeannie Chew, Danielle M. Cosio, Marka van Blitterswijk, Wing C. Lee, Rosa Rademakers, Kevin B. Boylan, Dennis W. Dickson, Leonard Petrucelli

**Affiliations:** 1 Department of Neuroscience, Mayo Clinic Florida, Jacksonville, FL 32224 USA; 2 Mayo Graduate School, Mayo Clinic College of Medicine, Rochester, MN 55905 USA; 3 Department of Pharmacology, Boston University School of Medicine, Boston, MA 02118 USA; 4 Department of Neurology, Mayo Clinic Florida, Jacksonville, FL 32224 USA

**Keywords:** Amyotrophic lateral sclerosis, Bidirectional transcription, C9ORF72, Expanded repeat, Frontotemporal dementia, Repeat-associated non-ATG translation, RNA foci

## Abstract

Frontotemporal dementia (FTD) and amyotrophic lateral sclerosis (ALS) are devastating neurodegenerative disorders with clinical, genetic, and neuropathological overlap. A hexanucleotide (GGGGCC) repeat expansion in a non-coding region of *C9ORF72* is the major genetic cause of both diseases. The mechanisms by which this repeat expansion causes “c9FTD/ALS” are not definitively known, but RNA-mediated toxicity is a likely culprit. RNA transcripts of the expanded GGGGCC repeat form nuclear foci in c9FTD/ALS, and also undergo repeat-associated non-ATG (RAN) translation resulting in the production of three aggregation-prone proteins. The goal of this study was to examine whether antisense transcripts resulting from bidirectional transcription of the expanded repeat behave in a similar manner. We show that ectopic expression of (CCCCGG)_66_ in cultured cells results in foci formation. Using novel polyclonal antibodies for the detection of possible (CCCCGG)_exp_ RAN proteins [poly(PR), poly(GP) and poly(PA)], we validated that (CCCCGG)_66_ is also subject to RAN translation in transfected cells. Of importance, foci composed of antisense transcripts are observed in the frontal cortex, spinal cord and cerebellum of c9FTD/ALS cases, and neuronal inclusions of poly(PR), poly(GP) and poly(PA) are present in various brain tissues in c9FTD/ALS, but not in other neurodegenerative diseases, including CAG repeat disorders. Of note, RNA foci and poly(GP) inclusions infrequently co-occur in the same cell, suggesting these events represent two distinct ways in which the *C9ORF72* repeat expansion may evoke neurotoxic effects. These findings provide mechanistic insight into the pathogenesis of c9FTD/ALS, and have significant implications for therapeutic strategies.

**Electronic supplementary material:**

The online version of this article (doi:10.1007/s00401-013-1192-8) contains supplementary material, which is available to authorized users.

## Introduction

An expanded GGGGCC hexanucleotide repeat within a non-coding region of the *C9ORF72* gene is the most common genetic cause of frontotemporal dementia (FTD) and amyotrophic lateral sclerosis (ALS), two devastating multisystem neurodegenerative disorders with significant genetic, neuropathological, and clinical overlap [11, 30]. FTD, a common form of early-onset dementia, is characterized clinically by abnormalities in behavior and language, whereas ALS is characterized by upper and lower motor neuron signs, which include weakness and muscle atrophy. FTD-like cognitive and behavioral impairments are also present in up to 50 % of ALS patients [16, 19, 27], and as many as half of FTD patients develop motor neuron dysfunction (MND) reminiscent of ALS [19].

The mechanisms by which the *C9ORF72* hexanucleotide repeat expansion causes “c9FTD/ALS” are not definitively known, but at least three pathogenic pathways may be at play. Several groups have shown that mRNA levels of certain *C9ORF72* variants are decreased in c9FTD/ALS [11, 15, 25, 30], suggesting loss of C9ORF72 function as a potential neurotoxic mechanism. In addition, RNA transcripts containing the expanded repeat may cause neurodegeneration by two means: through their accumulation into discrete structures in the nucleus, termed RNA foci, and by serving as a template for the synthesis of aberrantly expressed, aggregation-prone proteins by repeat-associated non-ATG (RAN) translation. Indeed, (GGGGCC)_exp_ RNA foci are observed in c9FTD/ALS [11], and the accumulation of neuronal inclusions composed of “c9RAN proteins” is a pathological hallmark of c9FTD/ALS [3, 25].

RNA foci are thought to cause cellular toxicity by sequestering specific RNA-binding proteins in a sequence-dependent manner, consequently disrupting their function [4]. In myotonic dystrophy type 1 (DM1), for instance, RNA foci formed of CAG·CTG repeat transcripts bind and inactivate the splicing factor muscleblind-like 1 protein (MBNL1) [23, 32, 33]. This sequestration of MBNL1 results in the mis-splicing of a subset of pre-mRNA targets that account for some of the characteristic features of disease [10, 20]. Transcripts of (GGGGCC)_exp_ that accumulate as nuclear foci in c9FTD/ALS may similarly sequester RNA-binding proteins and cause the misregulation of crucial downstream RNA targets leading to cellular dysfunction [24, 29, 38].

In addition to foci formation, transcripts of expanded repeats may be susceptible to RAN translation, an unconventional mode of translation that occurs across expanded repeat tracts despite the absence of an initiating codon. First described by Ranum and colleagues for expanded trinucleotide CAG·CTG repeats [39], transcripts of expanded CGG repeats [34], and the aforementioned GGGGCC repeats [3, 25] are now known to be RAN translated. Because RAN translation can occur in all possible reading frames, various products can be synthesized from a given transcript. Recently, we, as well as the Edbauer group and colleagues, independently reported that RAN translation of (GGGGCC)_exp_ RNA in c9FTD/ALS results in the production of poly(GP), poly(GA), and poly(GR) proteins [3, 25]. The presence of neuronal inclusions composed of these c9RAN proteins throughout the central nervous system is now considered pathognomonic of c9FTD/ALS [3, 25].

For many microsatellite expansion disorders, the expanded repeat is bidirectionally transcribed [4]. Detectable expression of both sense and antisense transcripts containing the hexanucleotide repeat in *C9ORF72* patients indicates that bidirectional transcription also occurs in c9FTD/ALS [25]. Consequently, not only do (GGGGCC)_exp_ transcripts form foci and undergo RAN translation, so too may (CCCCGG)_exp_ transcripts resulting from bidirectional transcription of the *C9ORF72* expanded repeat. The goal of this study was thus to examine whether (CCCCGG)_exp_-derived RNA foci and c9RAN translation proteins are present in c9FTD/ALS.

## Materials and methods

### Secondary structure prediction and model building of CCCCGG repeats

Folding of the RNA sequence comprising CCCCGG repeats of 10 (60 bases), 50 (300 bases), and 200 (1,200 bases) was carried out as previously described to determine secondary structure motifs [3]. The secondary structure prediction and modeling was built by examining the output from several RNA prediction packages: MFOLD, Sfold, Vienna RNA Package (RNAfold) [12, 17, 18, 21, 22, 31, 40, 41]. MFOLD utilizes a minimum free energy RNA structure prediction algorithm, Sfold utilizes statistical sampling of all possible structures, while Vienna Package has several options, including an minimum free energy calculation. Equivalent structures were given from each package for the ten repeat cases. MFOLD was used as the primary secondary structure prediction package for consistency across all models. Free energies were calculated for each secondary structure prediction at the level of decomposition (base pairing) and global energy [36]. MFOLD verifies each secondary structure prediction generated for valid structure [36]. MFOLD parameters include the following: (1) linear RNA sequence; (2) zero constraints, forces, or prohibitions on all bases allowing maximal sampling; (3) folding temperature of 37 °C; (4) physiological ionic conditions; (5) structure draw mode: untangle with loop fix; and (6) the remainder of MFOLD settings were set to default.

### Generation of antibodies

For each of the peptide antigens (C-Ahx-(PR)_8_-amide, C-Ahx-(GP)_8_-amide and C-Ahx-(PA)_8_-amide), two rabbits were immunized. Pre-immune serum from each rabbit was tested against peptide antigens and tissue from c9FTD/ALS cases by Western blot and immunohistochemistry, respectively, and confirmed negative. Antiserum was used directly or affinity purified before use.

### Meso Scale Discovery immunoassays

Peptides diluted in Tris-buffered saline (TBS) were added to duplicate wells (35 μl/well) of a 96-well MSD assay plate at final concentrations 0.1 μg/well. Following overnight incubation at 4 °C, wells were washed with TBS containing 0.2 % Tween 20 (TBSTw), and blocked with TBSTw+3 % non-fat milk. Antibody solution (25 μl/well) containing the indicated anti-PR, anti-GP, anti-PA, anti-GA or anti-GR antibodies (1:1,000) and SULFO-TAG™-rabbit secondary antibody (0.5 μg/ml, in blocking buffer) was added. Following a 2-h incubation and final washes, antibody binding to immobilized peptides was evaluated by adding MSD Read Buffer and measuring light emission at 620 nm upon electrochemical stimulation using the MSD Sector Imager 2400.

### Western blot analysis and immunofluorescence staining for antibody characterization

HEK293T cells were transfected with Lipofectamine™ 2000 with pEGFP-C1 vector only, or pEGFP-C1 (Clontech) plasmids into which oligonucleotides of five repeats of PR, GP, PA, GA or GR were inserted. For Western blotting, cell lysates collected 2 days post-transfection were resolved by 10 % Tris–Glycine SDS-PAGE (Invitrogen) and transferred to nitrocellulose membranes for probing with anti-PR, anti-GP, anti-PA, anti-GA or anti-GR, or with anti-GFP (Abcam, 1:2,000). For immunostaining, coverslips were fixed, permeabilized and blocked, then probed with the indicated antibodies followed by anti-rabbit-AF594 (Alexa Fluor) and Hoechst [3].

### Cloning of CCCCGG expression vectors

To generate the antisense (CCCCGG)_2_ and (CCCCGG)_66_ expression vectors, we first generated sense (GGGGCC)_2_ and (GGGGCC)_66_ expression vectors. Toward this end, genomic DNA from muscle or spleen from a *C9ORF72* expanded repeat carrier was used as a template in a nested PCR strategy using ThermalAce DNA Polymerase (Invitrogen) to amplify the (GGGGCC)_n_ repeat region, including 113 bp of 5′ and 99 bp of 3′ flanking sequence. The upstream primer used was 5′-AAGGAAGCTTAGTACTCGCTGAGGGTGAAC-3′; downstream primers used were 5′-GCTTGGATCCCCCACTCGCCACCGCCTG-3′ and 5′-GTCAGAGAAATGAGAGGGAAAG-3′. The PCR products were cloned into the pAG3 expression vector (kindly provided by Dr. T. Golde, University of Florida) using restriction sites *Hin*dIII and *Bam*HI. The pAG3 expression vector has a pcDNA3.0 backbone and a CMV-enhanced chicken B-actin promoter. The clones containing (GGGGCC)_2_ or (GGGGCC)_66_ were screened by colony PCR, and further verified by hairpin sequence analysis. The plasmids of (GGGGCC)_2_ or (GGGGCC)_66_ were digested using *Hin*dIII, and then the overhangs were filled-in using DNA Polymerase I (Klenow) Fragment. After purification, the fragments were digested with *Bam*HI. The inserts with (GGGGCC)_2_ or (GGGGCC)_66_, including the 5′ and 3′ flanking sequences, were re-cloned in the antisense orientation into a pAG3 expression vector using *Bam*HI and EcoRV to generate the (CCCCGG)_2_ and (CCCCGG)_66_ expression constructs. The sequence of antisense vectors was verified by hairpin sequence analysis. Note that the DNA sequence of the CCCCGG repeat is provided in Online Resource 1, which also highlights the regions included in the generation of pAG3-(CCCCGG)_2_ and pAG3-(CCCCGG)_66_ expression vectors.

### RNA fluorescence in situ hybridization of cultured cells expressing (CCCCGG)_n_ expression vectors

Evaluation of foci formation in HeLa cells transfected with (CCCCGG)_2_ or (CCCCGG)_66_ expression vectors was carried out by RNA fluorescence in situ hybridization (FISH). In brief, cells grown on glass coverslips were transfected with 0.5 μg of the (CCCCGG)_2_ or (CCCCGG)_66_ constructs. After 36 h, cells were fixed and permeabilized in 4 % paraformaldehyde + 20 % acetic acid + 2 mM ribonucleoside vanadyl complex (Sigma) for 10 min at room temperature. Cells were then washed with phosphate buffered saline treated with diethylpyrocarbonate (DEPC–PBS), and hybridized with denatured Cy3-conjugated (GGGGCC)_4_ probe (2 ng/μl) in hybridization buffer (50 % formamide, 10 % dextran sulfate, 0.1 mg/ml yeast tRNA, 2X saline–sodium citrate buffer (SSC), 50 mM sodium phosphate buffer) overnight at 37 °C. After washing once with 40 % formamide/1XSSC for 30 min at 37 °C, and twice with DEPC–PBS for 5 min at room temperature, nuclei were counterstained with Hoechst 33258 (1 μg/ml, Invitrogen) prior to mounting coverslips. Images were obtained on a Zeiss LSM 510 META confocal microscope.

### Western blot analysis of cultured cells expressing (CCCCGG)_n_ expression vectors

To determine whether ectopic expression of expanded CCCCGG transcripts leads to RAN translation, HEK293T cells were transfected with 5 μg of the (CCCCGG)_2_ or (CCCCGG)_66_ constructs. After 36 h, cells were harvested and washed with ice-cold PBS (pH 7.4), then cell pellets were lysed in buffer (50 mM Tris–HCl, pH 7.4, 300 mM NaCl, 1 % Triton-X-100, 5 mM EDTA, 2 % sodium dodecyl sulfate (SDS), plus phenylmethylsulfonyl fluoride (PMSF) and both a protease and phosphatase inhibitor mixture). After centrifugation at 16,000*g* for 20 min at 4 °C, the supernatant was collected and protein concentration determined by BCA assay. For Western blot analysis, samples were prepared in Laemmli’s buffer, heated for 5 min at 95 °C, and equal amounts of protein (30 μg) were loaded into Novex^®^ 10–20 % Tricine gels. After transfer, blots were blocked with 5 % non-fat dry milk in TBST for 1 h, and then incubated with the purified anti-PR, anti-GP or anti-PA (1:1,000), or mouse monoclonal GAPDH antibody (1:10,000, Biodesign) overnight at 4 °C. Membranes were washed then incubated with anti-species horseradish peroxidase-linked secondary antibodies (1:5,000; Jackson ImmunoResearch) for 1 h. Protein expression was visualized by enhanced chemiluminescence treatment and exposure to film.

### Human case material

All cases examined in this study were selected from a series of autopsied brains submitted to the neuropathology laboratory at Mayo Clinic in Jacksonville. The sources of this case material include the Mayo Clinic Florida ALS Center (*n* = 11), referral to the Parkinson disease brain bank (*n* = 7), the State of Florida Alzheimer’s Disease Initiative (*n* = 2), Florida Alzheimer’s Disease Research Center (*n* = 1) and CurePSP/Society of Progressive Supranuclear Palsy brain bank (*n* = 1). The presence or absence of *C9ORF72* repeat expansion was determined using frozen cerebellar tissue from the right hemibrain and a previously described repeat-primed polymerase chain reaction (PCR) method [11]. In addition, repeat length was estimated using Southern blotting techniques as previously described [35] (Table 1). In brief, 7–10 μg of high-quality genomic DNA extracted from frozen frontal cortex and cerebellum was digested with *XbaI*, and electrophoresed in a 0.8 % agarose gel. DNA was then transferred to a positively charged nylon membrane (Roche), cross-linked, and subsequently hybridized with a DIG-labeled probe. Expansions were visualized with anti-DIG antibody (Roche) and CDP-star substrate (Roche) on X-ray film after multiple exposures. The most abundant expansion sizes were estimated using AlphaEaseFC (Alpha Innotech) based on their position relative to DNA molecular weight markers.

**Table 1:** *C9ORF72* antisense transcript RAN translation cohort

Case #	*C9ORF72* mutation yes/no	Pathological diagnosis	Age at death	Sex	Repeat size		Hippocampal burden			Cerebellar burden		
					Frontal cortex	Cerebellum	PA	PR	GP	PA	PR	GP
1	Y	FTLD-TDP	66	M	16.0	12.5	±	+	+++	±	+	+++
2	Y	FTLD-TDP	71	M	25.2	11.4	±	±	++	±	±	++
3	Y	FTLD-TDP	86	M	50.0	17.5	+	±	+++	±	±	+++
4	Y	FTLD-MND	68	M	25.6	12.8	±	±	++	±	±	+++
5	Y	FTLD-MND	70	M	38.4	10.1	+	+	+++	+	+	+++
6	Y	FTLD-MND	61	F	35.6	13.7	+	+	+++	±	±	+++
7	Y	ALS	53	M	46.8	14.9	+	+	+++	+	+	+++
8	Y	ALS	49	F	23.8	10.0	+	+	+++	±	±	+++
9	Y	ALS	41	F	22.3	10.9	+	+	+++	+	+	+++
10	N	FTLD-TDP	88	F	−	−	−	−	−	−	−	−
11	N	FTLD-TDP	65	M	−	−	−	−	−	−	−	−
12	N	FTLD-TDP	83	F	−	−	−	−	−	−	−	−
13	N	FTLD-MND	64	M	−	−	−	−	−	−	−	−
14	N	FTLD-MND	72	F	−	−	−	−	−	−	−	−
15	N	FTLD-MND	66	F	−	−	−	−	−	−	−	−
16	N	ALS	60	M	−	−	−	−	−	−	−	−
17	N	ALS	61	F	−	−	−	−	−	−	−	−
18	N	ALS	53	F	−	−	−	−	−	−	−	−
19	N	HD	80	F	−	−	−	−	−	−	−	−
20	N	HD	70	F	−	−	−	−	−	−	−	−
21	N	Kennedy’s	80	M	−	−	−	−	−	−	−	−
22	N	SCA3	53	M	−	−	−	−	−	−	−	−

− absent, ± sparse (<5 inclusions), + mild, ++ moderate, +++ severe

### Fluorescence in situ hybridization and immunofluorescence staining of human tissue

Formalin-fixed, paraffin-embedded frontal cortex, spinal cord, and cerebellum sections were cut at a 5-μm thickness and mounted on glass slides, then subjected to RNA FISH followed by immunofluorescence staining. For FISH, slides were deparaffinized and rehydrated, incubated with pepsin (4 mg/ml in 0.9 % NaCl, pH 1.5) for 20 min at 37 °C, rinsed in water, then immersed in ice-cold 20 % acetic acid for 90 s, prior to dehydration. A Cy3-tagged (GGGGCC)_4_ probe (IDT) which hybridizes to the expanded CCCCGG repeat was applied to the tissue, which was then sealed under a coverslip. Prior to use, the probe was diluted to 5 ng/μl in hybridization buffer (10 % dextran sulfate, 50 % formamide, 2XSSC, 50 mM sodium phosphate buffer, 10 ng/ml tRNA pH 7.0), then heated at 80 °C for 10 min, placed on ice for 5 min, then heated at 37 °C for 10 min. Alternatively, to detect foci formed of (GGGGCC)_exp_ RNA, a TYE563-labeled LNA probe (5′TYE563-CCCGGCCCCGGCCCC-3′TYE563; Exiqon, Inc) was applied to the tissue. This probe was diluted to 0.4 ng/ml in hybridization buffer (10 % dextran sulfate, 50 % formamide, 2XSSC, 50 mM sodium phosphate buffer, 10 ng/ml tRNA, pH 7.0), then heated at 80 °C for 75 s, according the manufacturer’s instructions. Following a 2-day hybridization at 37 or 55 °C for the antisense and sense probe, respectively, coverslips were removed and slides were washed: once in 2X SSC, three times in 50 % formamide/2X SCC at 37 °C, and three times in 1X SSC at 37 °C. Slides were subsequently subjected to immunofluorescence staining: slides were blocked with DAKO Serum-Free Protein Block and then incubated with anti-GP (1:3,000), choline acetyltransferase antibody (ChAT; 1:200; Chemicon AB144P), microtubule-associated protein 2 antibody (MAP2; 1:750, clone AP-20) or glial fibrillary acidic protein antibody (GFAP; 1:500, clone GA-5) overnight at 4 °C. The following day, slides were incubated with an Alexa Fluor 488-conjugated secondary antibody (1:500, Molecular Probes) for 1.5 h at room temperature. Slides were then treated with a solution of Sudan Black for 2 min to block auto-fluorescence, and coverslipped using Vectashield-DAPI mounting medium (Vector Laboratories).

Imaging was performed using a Zeiss Axio Imager Z1 microscope to visualize foci and poly(GP) inclusions. Although it is difficult to distinguish binding of the probe to an individual RNA transcript given that the fluorescence resulting thereof is not sufficiently strong to yield a signal above background, the FISH method employed is well suited to examine nuclear foci. The presence of multiple labeled transcripts in a compact location, as is the case for foci, results in a highly distinct, punctate fluorescent signal much brighter than background. Note that, to validate specificity of the probes targeting GGGGCC and CCCCGG RNA, FISH was carried out on frontal cortex and cerebellar tissue from ALS, frontotemporal lobar degeneration (FTLD), and FTLD-MND patients with normal *C9ORF72* repeat length. In addition, a (CAGG)_6_ probe targeting the CCTG repeat was tested and shown also to be negative.

To determine what percentage of affected cells (i.e., cells having either foci or a poly(GP) inclusion) have *both* nuclear foci and poly(GP) pathology, we analyzed frontal cortex and cerebellar sections of four c9FTD/ALS cases co-stained for either sense or antisense foci and poly(GP) inclusions. For each section, we examined 25 cells with inclusions by fluorescence microscopy, and then changed the excitation filter to determine whether they also had foci; we then examined 25 cells with foci and determined whether they had inclusions. In this manner, a total of 50 cells were examined per section. For each group (Group 1—frontal cortex probed for antisense foci and poly(GP) inclusions; Group 2—cerebellum probed for antisense foci and poly(GP) inclusions; Group 3—frontal cortex probed for sense foci and poly(GP) inclusions; Group 4—cerebellum probed for sense foci and poly(GP) inclusions), the percentage of cells having both foci and inclusions was calculated and compared by two-way ANOVA. In addition, we compared the frequency of sense and antisense foci in the frontal cortex of the four examined cases. For each section, the number of cells with foci and the number of total cells were counted in 12 randomly selected, non-overlapping fields from layers I–III. The average percentage of cells with antisense foci among the four cases was compared to the average percentage of cells with sense foci by paired, two-tailed *t* test.

### Immunohistochemistry

For immunohistochemical analysis, 9 cases exhibiting an expanded *C9ORF72* repeat (3 FTLD-TDP, 3 FTLD-MND, 3 ALS) were matched with 13 negative control cases that lacked the expanded repeat, including 3 FTLD-TDP, 3 FTLD-MND, 3 ALS, and 4 cases with CAG repeat disorders, including 2 cases with Huntington’s disease, 1 case with Kennedy’s disease, and 1 case with spinocerebellar ataxia type 3 (Table 1). For the 9 c9FTD/ALS cases and the 9 FTD/ALS matched controls, immunohistochemistry was performed on formalin-fixed paraffin-embedded tissue from the motor cortex, hippocampus (including temporal cortex), basal forebrain (including amygdala), thalamus (at the level of the subthalamic nucleus), medulla, and cerebellum. For the four trinucleotide repeat disorders, sections from the basal forebrain (2 Huntington’s disease cases), medulla (Kennedy’s disease case) or pons (spinocerebellar ataxia type 3 case) were used. Five-micron-thick tissue sections were cut from paraffin blocks, mounted on charged glass slides, and allowed to dry overnight at 65 °C. The following day, slides were deparaffinized and rehydrated in serial washes in xylene and alcohol before steaming the slides for 30 min in 1X Tris–EDTA (pH 9) buffer solution. Immunohistochemistry was performed using the Dako Autostainer and the Dako EnVision™ + Rabbit (DAB) kits. Immunostaining was performed with the following antibodies: anti-PA (Rb8604, 1:2,500), anti-GP (Rb7379, 1:10,000), and anti-PR (Rb8736, affinity purified, 1:100). Following immunohistochemistry, slides were counterstained with Lerner’s hematoxylin, dehydrated, and coverslipped. All imaging was conducted using the Zeiss Axio Imager Z1 microscope.

## Results

### Characterization of antibodies for the detection of c9RAN proteins produced by RAN translation of (CCCCGG)_exp_ RNA

**Fig. 1:**
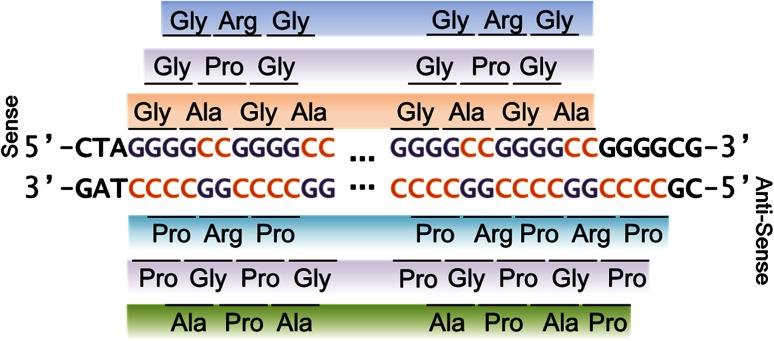
Schematic representation of the possible proteins generated by RAN translation of expanded GGGGCC and CCCCGG repeats in all possible reading frames

The RNA structure of expanded repeats is believed to influence their susceptibility to RAN translation [9, 39]. We had previously reported that (GGGGCC)_exp_ RNA is predicted to form imperfect hairpins and is RAN translated [3]. Using methodologies for generating secondary structure predictions, we thus sought to determine whether (CCCCGG)_exp_ RNA is predicted to have a similar structure and also be prone to RAN translation. It should be noted that, while the reverse complement of the 5′-GGGGCC-3′ repeat is 5′-GGCCCC-3′, there are 4 Gs downstream of the last sense GGGGCC repeat, thus making the first antisense repeat CCCCGG (Fig. 1, Online Resource 1). The major RNA secondary structure prediction for 10 antisense CCCCGG repeats or 10 sense GGGGCC repeats is stable imperfect hairpins, but the GGGGCC repeat structure has a lower composite global energy (Δ*G* = −40.8 kcal/mol), and therefore greater stability, than the antisense CCCCGG repeats (Δ*G* = −35.3 kcal/mol) (Online Resource 2). Nonetheless, both GGGGCC and CCCCGG repeats become successively more stable as repeat length increases (Online Resource 2b). Given these observations, the CCCCGG repeat, like its GGGGCC counterpart, may be RAN translated. To determine whether this is indeed the case, two independent rabbit polyclonal antibodies were generated against each of the products that could be produced from RAN translation of (CCCCGG)_exp_ transcripts in the three alternate reading frames: poly(PR), poly(PG), and poly(PA) (Fig. 1). Given that poly(PG) [aka poly(GP)] proteins can be translated from both sense and antisense transcripts of the *C9ORF72* repeat expansion (Fig. 1), “GP” is henceforth used when referring to these proteins.

**Fig. 2:**
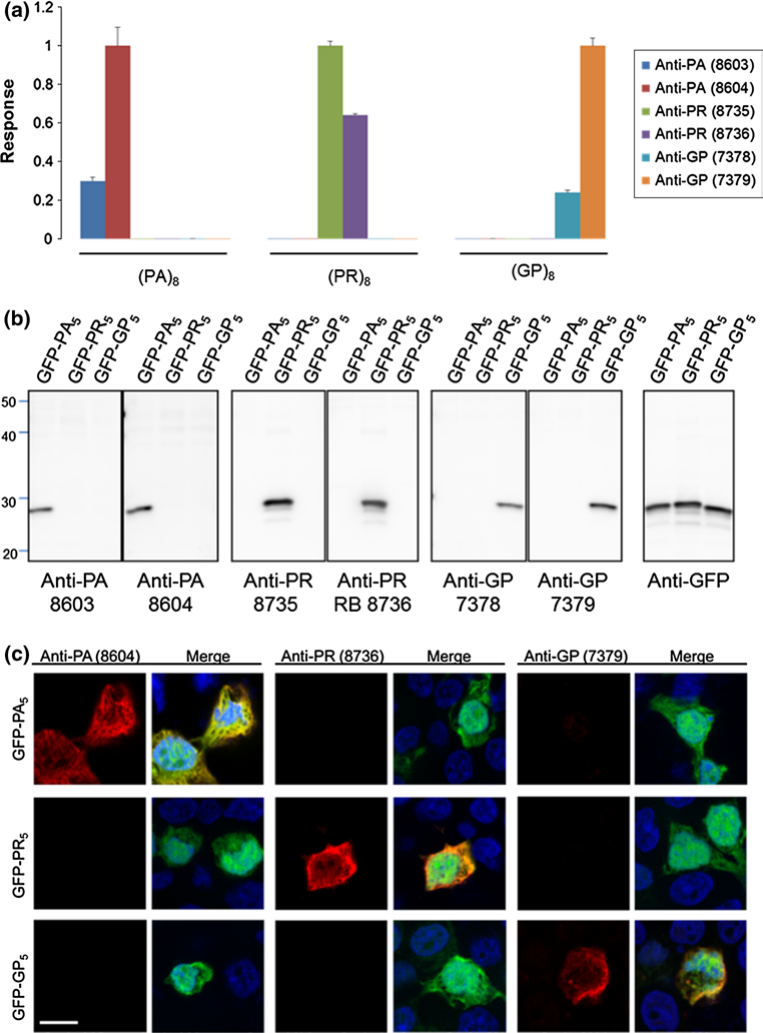
Antibody characterization for c9RAN proteins. **a** The immunoreactivity of antibodies to c9RAN proteins towards (PA)_8_, (PR)_8_ and (GP)_8_ peptides was measured by adsorbing peptides onto carbon electrodes in 96-well MSD plates, and co-incubating wells with anti-PA, anti-PR or anti-GP antibodies, and a SULFO-tagged anti-rabbit secondary antibody. Antibody binding to respective peptides was quantified by measuring the intensity of emitted light upon electrochemical stimulation of the plate using the MSD Sector Imager 2400. For each pair of antibodies, binding responses were normalized to the signal of the antibody showing the highest binding to its respective antigen. *Error bars* indicate standard deviations from duplicate wells. **b** Western blot analysis of lysates from HEK293T cells transfected to express enhanced GFP-tagged (PA)_5_, (PR)_5_ or (GP)_5_. Blots were probed with the indicated antibodies. **c** Immunofluorescence staining of HEK293T cells transfected to express the indicated enhanced GFP (*green*)-tagged peptides using anti-PA, anti-PR or anti-GP (*red*) antibodies. Nuclei are stained with Hoechst (*blue*). *Scale bar* 10 μm. Note that similar studies were conducted to test potential cross-reactivity of antibodies to poly(GA) and poly(GR), as shown in Online Resource 3

Specificity of anti-PR, anti-GP and anti-PA antibodies toward their respective antigen was evaluated by quantitative electrochemiluminescent immunoassays. Measurement of antibody binding to immobilized (PR)_8_, (GP)_8_ or (PA)_8_ peptides showed that all antibodies bound only their respective antigen (Fig. 2a). Consistent with these findings, anti-PR, anti-GP and anti-PA antibodies respectively detected exogenously expressed enhanced GFP-tagged (PR)_5_, (GP)_5_ or (PA)_5_ in HEK293T cells, as assessed by Western blot of cell lysates (Fig. 2b), and immunofluorescence staining of cells (Fig. 2c). Anti-PR, -GP and -PA antibodies were additionally tested against poly(GA) and poly(GR) peptides, which represent c9RAN proteins produced from the sense transcript (Fig. 1). All antibodies but one detected only their respective antigen, as assessed by immunoassay, Western blot and immunofluorescence staining of cultured cells (Online Resource 3). Anti-PR (7378), however, did show modest cross-reactivity with the GR peptide by immunoassay, but such cross-reactivity was not observed by Western blot or immunofluorescence staining (Online Resource 3).

### Exogenous (CCCCGG)_exp_ transcripts are subject to RAN translation in cultured cells

**Fig. 3:**
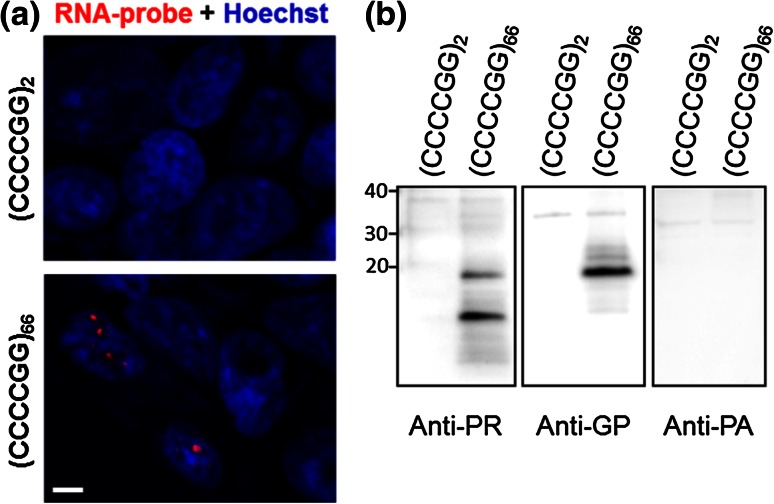
Expression of expanded CCCCGG repeats in cultured cells leads to foci formation and expression of c9RAN proteins. **a** HEK293T cells transfected to express (CCCCGG)_2_ or (CCCCGG)_66_ were subjected to RNA fluorescence in situ hybridization using a probe against CCCCGG repeat transcripts. Note the foci (*red*) in Hoechst-stained nuclei (*blue*) of (CCCCGG)_66_-expressing cells, but not (CCCCGG)_2_-expressing cells. *Scale bar* 5 μm. **b** Western blot analysis of lysates from (CCCCGG)_n_-expressing cells shows that poly(PR) and poly(GP) proteins, but not poly(PA) proteins, are expressed in cells transfected with (CCCCGG)_66_. No c9RAN protein was detected in control cells expressing non-expanded (CCCCGG)_2_

Having confirmed specificity of these novel antibodies toward potential (CCCCGG)_n_ c9RAN proteins, they were then used to evaluate whether poly(PR), poly(GP) or poly(PA) proteins can indeed be RAN translated from expanded CCCCGG repeats. To address this question, a (CCCCGG)_66_ expression vector having no initiating ATG start codon upstream of the repeats was generated for the transfection of cultured cells. As shown in Fig. 3a, expression of (CCCCGG)_66_, but not of non-expanded (CCCCGG)_2_, led to the formation of nuclear foci, as assessed by RNA FISH using a Cy3-tagged (GGGGCC)_4_ probe targeting the CCCCGG repeat. Ectopic expression of (CCCCGG)_66_ also resulted in the synthesis of poly(GP) and poly(PR) proteins, but not poly(PA), as assessed by Western blot of cell lysates (Fig. 3b). These c9RAN proteins were not observed in cells expressing (CCCCGG)_2_ suggesting that their formation is repeat length-dependent (Fig. 3b).

### Nuclear foci of (CCCCGG)_exp_ transcripts are present in brain and spinal cord of c9FTD/ALS patients

**Fig. 4:**
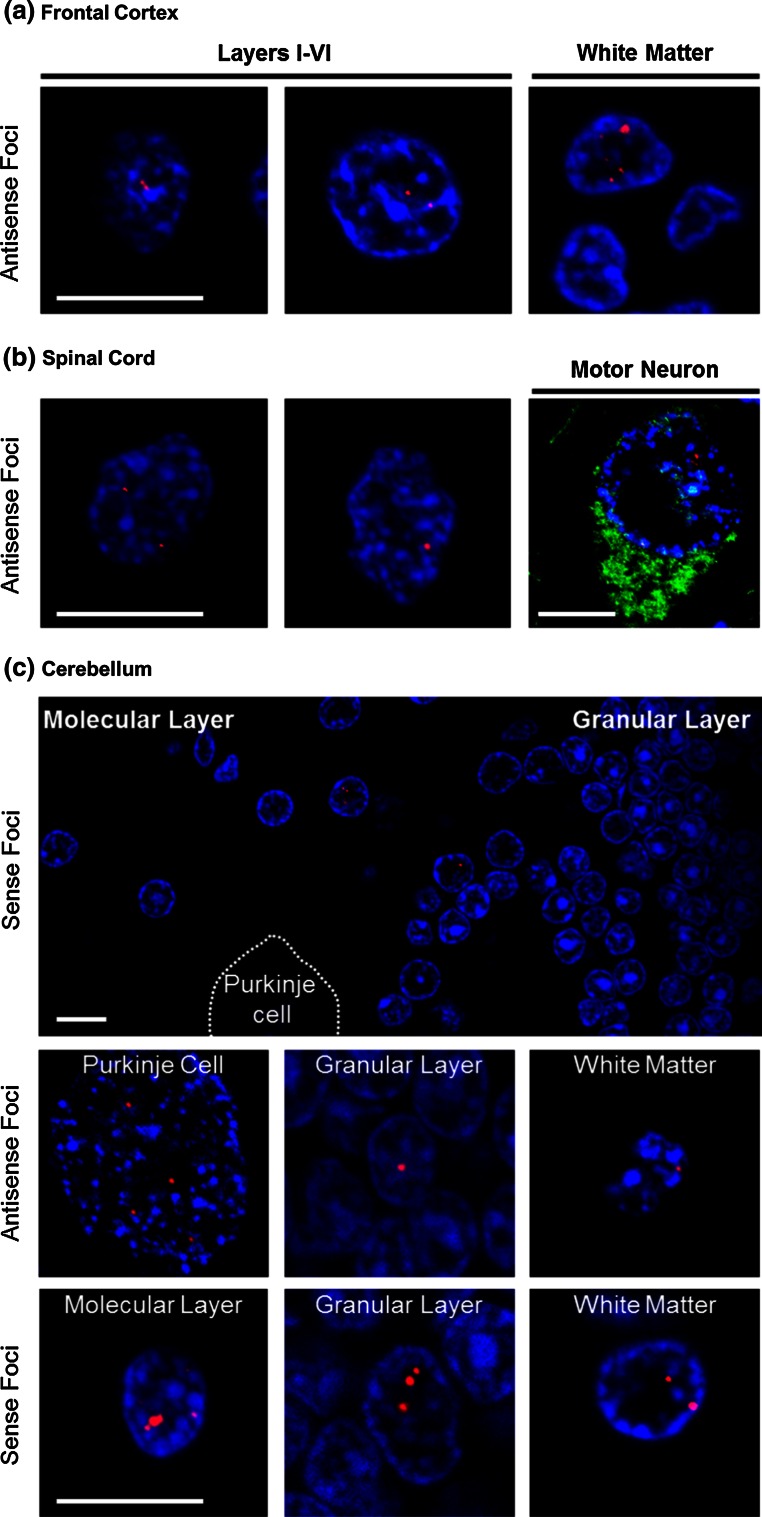
*C9ORF72* hexanucleotide repeat transcripts form nuclear RNA foci in frontal cortex, spinal cord and cerebellum in c9FTD/ALS. Fluorescence in situ hybridization (FISH) of c9FTD/ALS frontal cortex (**a**) and c9ALS spinal cord (**b**) tissue using a probe against the CCCCGG repeat transcripts shows RNA foci (*red*) in the nucleus (stained with DAPI, *blue*) of cells. In (**b**), note that foci are observed in motor neurons that stain positively for ChAT. **c** Cerebellar sections of c9FTD/ALS cases were subjected to FISH using a probe against CCCCGG repeat transcripts or GGGGCC repeat transcripts. In most instances, foci-bearing cells within the cerebellum were found in proximity to the Purkinje cell layer separating the molecular and granular layers. However, RNA foci were also observed in cells of the molecular layer, deep within the granular layer, in Purkinje cells, and in cells within the white matter. *Scale bars* 10 μm

It was previously shown that transcripts of the expanded GGGGCC repeat accumulate as nuclear RNA foci in the frontal cortex and spinal cord of c9FTD/ALS patients [11]. To determine whether expression of antisense transcripts of (CCCCGG)_exp_ similarly result in foci formation in human tissue, FISH was performed on paraffin-embedded frontal cortex and spinal cord sections from c9ALS, c9FTLD or c9FTLD-MND patients using the Cy3-(GGGGCC)_4_ probe, the specificity of which was verified using sections from affected patients with normal *C9ORF72* repeat lengths. Cells harboring (CCCCGG)_exp_ nuclear foci were observed throughout all layers of the frontal cortex, as well as in the white matter, of c9ALS, c9FTLD and c9FTLD-MND patients, with the number of foci in affected cells ranging from few (e.g., 1–2) to many (>10), and with the size of foci being variable (Fig. 4a). Likewise, (CCCCGG)_exp_ nuclear foci were present in cells of the spinal cord, including ChAT-immunopositive motor neurons (Fig. 4b).

**Fig. 5:**
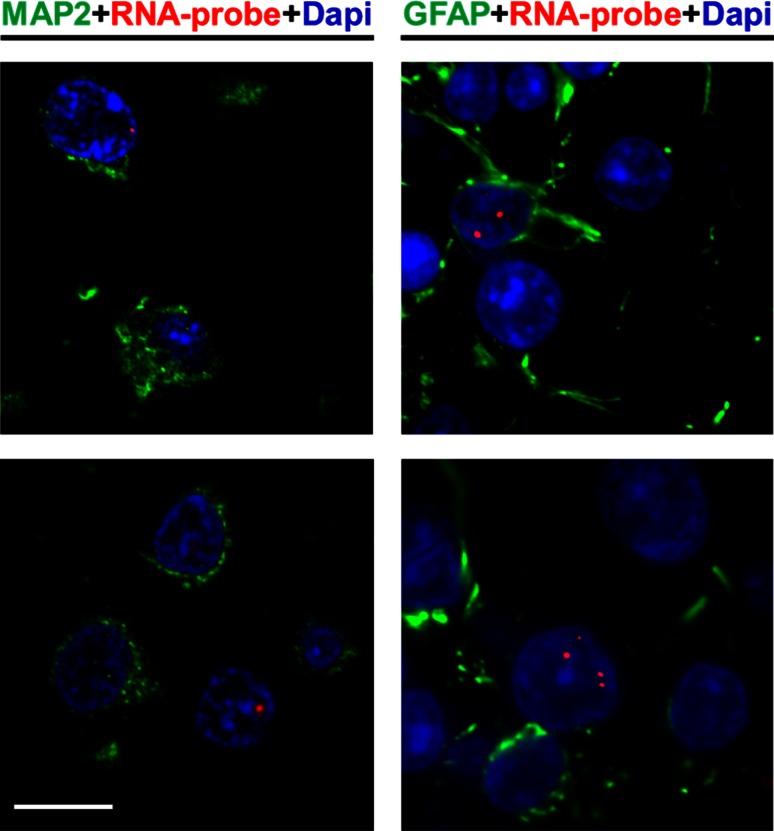
Nuclear RNA foci are present in both neurons and glia. Fluorescence in situ hybridization of c9FTD/ALS cerebellar tissue using a probe against the GGGGCC repeat was followed by immunofluorescence staining with the neuronal marker, MAP2, or the astrocytic marker, GFAP. Note that nuclear RNA foci are present in MAP2-positive and MAP2-negative cells, as well as GFAP-positive and GFAP-negative cells. *Scale bar* 10 μm

Compared to FTD and ALS cases lacking the *C9ORF72* expansion, c9FTD/ALS is marked by abnormal cerebellar pathology, such as inclusions composed of (GGGGCC)_exp_ RAN proteins [3, 25], as well as inclusions negative for TDP-43, but immunopositive for p62, ubiquitin, and various ubiquitin-binding proteins [1, 28]. Therefore, we evaluated whether (CCCCGG)_exp_ RNA foci, as well as (GGGGCC)_exp_ RNA foci, are present in the cerebellum of *C9ORF72* expanded repeat carriers. Multiple cells with RNA foci composed of sense or antisense transcripts were detected, and these were most often observed in proximity to the Purkinje cell layer separating the granular and molecular layers (Fig. 4c). Foci were also occasionally present in cells of the molecular layer, deep within the granular layer, in Purkinje cells, and in cells within the white matter (Fig. 4c). Post-FISH immunofluorescent staining with MAP2, a neuron marker, and GFAP, an astrocyte marker, confirmed that nuclear foci were present in both cell populations (Fig. 5). These findings indicate that, in addition to the frontal cortex and spinal cord, RNA foci resulting from the hexanucleotide repeat expansion in *C9ORF72* accumulate in the cerebellum.

### Inclusions of (CCCCGG)_exp_ c9RAN proteins are present in c9FTD/ALS

**Fig. 6:**
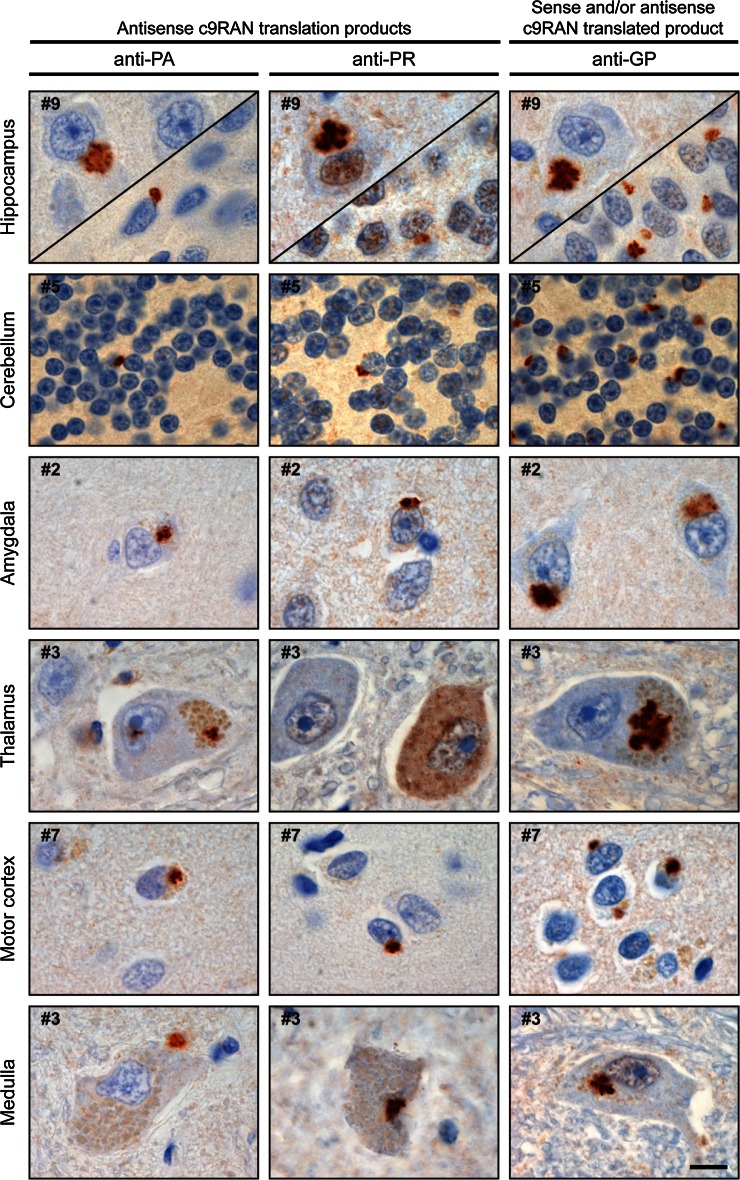
Antisense c9RAN proteins in human post-mortem tissue. Immunohistochemistry reveals poly(PA)- [*left column*], poly(PR)- [*middle column*], and poly(GP)- [*right column*] reactive lesions throughout the central nervous system, including the hippocampus (endplate-CA3 on the *top left*, dentate fascia on the *bottom right*), cerebellum, amygdala, thalamus, motor cortex (layers 2–3), and medulla (inferior olivary nucleus). Lesions are often neuronal cytoplasmic inclusions (NCI) with a star-shaped morphology, but can also appear as dense NCI, small neuronal intranuclear inclusions, or diffuse neuronal “pre-inclusions”. Anti-GP, which detects poly(GP) proteins that can be made from both sense and antisense transcripts of the *C9ORF72* expanded repeat, reveal greater pathologic burden compared to the anti-PA and PR antibodies. Case numbers correspond to c9FTD/ALS cases in Table 1. *Scale bar* 10 μm

Next, the presence of c9RAN proteins synthesized from the antisense repeat was analyzed in human brain tissue. Immunohistochemistry with the anti-PA and anti-PR antibodies revealed sparse neuronal cytoplasmic inclusions in all sections examined from the nine c9FTD/ALS cases. Lesion burden was greatest in the hippocampus (dentate fascia and hippocampus proper), lesser in the motor cortex, temporal cortex, amygdala, as well as thalamus, and lowest in the cerebellum and medulla (Fig. 6). Many of the lesions were of the characteristic “star-shaped” morphology. Immunohistochemistry with the anti-GP antibody revealed abundant pathology in all regions that paralleled the pathologic burden and distribution previously described with C9RANT antibodies [3]. Semi-quantitative analysis of anti-GP, anti-PA or anti-PR immunoreactive-inclusion burden in the hippocampus and cerebellum is shown in Table 1. Neuronal c9RAN protein inclusions were not seen in the nine FTD/ALS matched control cases, or in the four trinucleotide repeat disorder cases.

### RNA foci and poly(GP)-inclusions infrequently coexist in the same cell

**Fig. 7:**
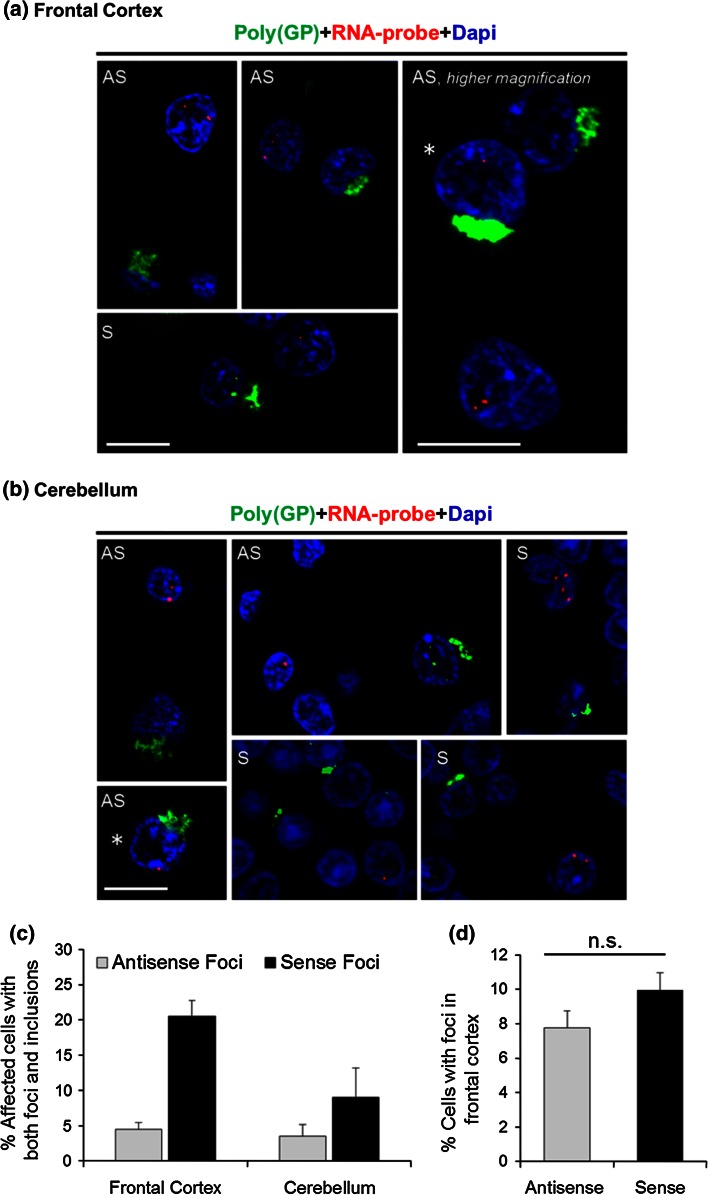
Nuclear RNA foci and poly(GP) inclusions are seldom observed in the same cell. Fluorescence in situ hybridization of frontal cortex and cerebellar tissue of c9FTD/ALS cases using a probe against sense and antisense *C9ORF72* hexanucleotide repeat transcripts was followed by immunofluorescence staining to detect poly(GP) inclusions, which may result from RAN translation of both sense and antisense transcripts. Though infrequent, both foci and poly(GP) inclusions can co-occur in the same cell (cells indicated by an *asterisk* in **a** and **b**). *Scale bars* 10 μm. *AS* antisense foci, *S* sense foci. **c** To determine the percentage of cells having both foci and poly(GP) inclusions in the frontal cortex and cerebellum, quantitative analysis was undertaken on four cases co-stained for poly(GP) inclusions and either sense or antisense foci. For each section, we examined 25 cells with inclusions and determined whether they had foci, and examined 25 cells with foci and determined whether they had inclusions, for a total of 50 cells per section. Data are presented as mean ± SEM, *n* = 4. The brain region sampled (frontal cortex vs. cerebellum, *P* = 0.0292) and foci type (antisense vs. sense, *P* = 0.0011) both significantly affect the percentage of cells having foci and inclusions, as assessed by two-way ANOVA. To determine the percentage of cells with antisense and sense foci in the frontal cortex, the number of cells with foci and the total number of cells were counted in 12 randomly selected, non-overlapping fields for each case. Data are presented as mean ± SEM, *n* = 4. No significant difference between the percentage of cells with sense or antisense foci was detected, as assessed by paired two-tailed *t* test (*P* = 0.1916) (**d**). *n.s.* not significant

The findings above, together with previous studies [2, 3, 11, 25], establish that (GGGGCC)_exp_ and (CCCCGG)_exp_ transcripts form nuclear RNA foci and undergo RAN translation; nonetheless, the relationship between these two events remains unclear. The presence of foci could be indicative of high levels of (GGGGCC)_exp_ and (CCCCGG)_exp_ transcripts, thus increasing the possibility of RAN translation. Conversely, the formation of nuclear foci may sequester these transcripts away from translational machinery, thus decreasing the likelihood of RAN translation. Given that both foci formation and RAN translation are potentially pathogenic, we examined the relationship between foci and inclusions composed of poly(GP), which is RAN translated from both sense and antisense transcripts. To this end, sections of cerebellar and frontal cortex from c9ALS, c9FTLD and c9FTLD-MND patients were subjected to FISH for the detection of (CCCCGG)_exp_ or GGGGCC_exp_ RNA foci, followed by immunofluorescence staining for poly(GP). Remarkably, while foci and anti-(GP)-immunoreactive inclusions were occasionally present in the same cell, the majority of affected cells had only RNA foci or only poly(GP) inclusions (Fig. 7a, b). To determine what percentage of cells have both foci and poly(GP) inclusions in the frontal cortex and cerebellum, quantitative analysis was undertaken on four cases co-stained for poly(GP) inclusions and either sense or antisense foci. For each section, we examined 25 cells with inclusions and determined whether they had foci, and examined an additional 25 cells with foci and determined whether they had inclusions. Shown in Fig. 7c is the percentage of affected cells that had both foci and inclusions. Note that the brain region sampled (frontal cortex vs. cerebellum) and foci type (antisense vs. sense) both significantly affect the percentage of cells having both foci and inclusions, as assessed by two-way ANOVA (Fig. 7c). The apparent increase in the percentage of cells having both inclusions and sense foci, compared to the percentage of cells having both inclusions and antisense foci, is not likely caused by increased frequency of sense foci. In the frontal cortex, where the difference between sense and antisense foci co-occurring with poly(GP)-inclusions is most pronounced, there was no difference in the percentage of cells with sense or antisense foci in the cases examined (Fig. 7d).

## Discussion

The discovery of new neuropathologic features specific to c9FTD/ALS, namely the formation of RNA foci and the production of c9RAN proteins resulting from the synthesis of antisense transcripts of the expanded *C9ORF72* repeat, provides additional insight into the pathobiology of c9FTD/ALS. Through the production of both sense and antisense expanded repeat RNA and five distinct c9RAN proteins, the repeat expansion leads to the production of seven potentially toxic biomolecules.

To determine whether antisense transcripts of the expanded *C9ORF72* repeat are RAN translated, we generated novel rabbit polyclonal antibodies for the detection of poly(GP), poly(PR) and poly(PA) proteins. Examination of RAN translation in cultured cell models showed that poly(GP) and poly(PR) proteins were synthesized in (CCCCGG)_66_-expressing cells, but not in cells expressing only two CCCCGG repeats. That poly(PA) proteins were not detected in (CCCCGG)_66_-expressing cells may be because a crucial upstream sequence necessary for translation of poly(PA) peptides is missing from the (CCCCGG)_66_ expression vector. Alternatively, it may be due to the fact that RAN translation is repeat length-dependent, with different reading frames having different length thresholds [9, 39]. Nonetheless, our immunohistochemical analysis of c9RAN proteins in human tissue indicates either this sequence is present, or the repeat length threshold is met, in c9FTD/ALS patients.

Because poly(GP) proteins can be synthesized from sense and antisense transcripts of the expanded *C9ORF72* repeat, their exact origin is not definitely known. Yet, the presence of poly(PA) and poly(PR) neuronal inclusions in post-mortem c9FTD/ALS brain tissue is indicative of RAN translation of the antisense transcript. These inclusions are specific to c9FTD/ALS cases, not being found in matched FTD/ALS controls lacking the *C9ORF72* expanded repeat, or in other repeat disorders. We did note a difference between poly(PA) and poly(PR) pathology in comparison to poly(GP) pathology, the latter being markedly more frequent. For example, poly(GP) pathology is extensive in granule cells and primary neurons of the cerebellum, but cerebellar poly(PA) and poly(PR) inclusions are sparse in these same populations.

While we cannot rule-out the possibility that poly(GP) inclusions appear more abundant because of differences in antibody affinities, their increased frequency may be due to the fact that poly(GP) proteins are synthesized from both sense and antisense transcripts. In addition, the antisense transcript may be less efficiently translated than the sense transcript. CCCCGG repeats are predicted to fold into an imperfect hairpin akin to what we have previously shown for GGGGCC repeats [3]. However, compared to the GGGGCC repeat, which has an optimal organization to maximize base pairing, the CCCCGG repeat has fewer base pairs and more frequent sets of four unpaired C nucleotides within the stem, and thus relies more on base stacking effects. While the stability of both CCCCGG and GGGGCC repeat transcripts increases with increasing repeat length, the CCCCGG repeat is predicted to be less stable than its GGGGCC counterpart, which may decrease its susceptibility to RAN translation. It should be mentioned that two previous studies report that r(GGGGCC) repeats form an intramolecular G-quadraplex structure [13, 29]. As has been suggested for other quadruplexes [8] and r(CGG) repeats, the r(GGGGCC) repeat, and perhaps the r(CCCCGG) repeat, may adopt two conformations that are in equilibrium: an extended hairpin structure and a quadruplex.

Another possible explanation for the higher frequency of poly(GP) inclusions in c9FTD/ALS may be due to ATG-initiated translation. While no sequence has been reported for the antisense transcript, resorting to analysis of the genomic DNA consensus sequence for *C9ORF72* has revealed no ATG codons for sense transcript-derived c9RAN proteins, or for the antisense transcript-derived poly(PA) protein. However, one and three potential ATG start codons were found in the antisense poly(PR) and poly(GP) frames, respectively (Online Resource 1). Nonetheless, whether ATG codons are in fact present in RNA transcripts is not yet known, and our cell culture data provide evidence that poly(PR) and poly(GP) proteins can be translated from (CCCCGG)_exp_ in the absence of an ATG initiation site.

Whether aberrant expression of c9RAN proteins, and the inclusions formed thereof, influence disease pathogenesis remains to be elucidated. Nevertheless, the formation of abnormal proteinaceous inclusions is associated with neurotoxicity in various neurodegenerative diseases. The inclusions may sequester other proteins causing loss of function, impair/overwhelm protein degradation systems, displace cytoplasmic organelles, and may themselves have neurotoxic properties. It is noteworthy that polyA and polyG proteins synthesized by RAN translation of expanded CAG·CTG repeats accumulate in disease-relevant tissues of patients with spinocerebellar ataxia type 8 and DM1, and that their expression in cultured cells is sufficient to cause apoptotic cell death [26]. In addition, in both DM1 patients and in mice expressing (CUG)_exp_, polyG aggregates co-localize with caspase-8, an early indicator of polyG-induced apoptosis [26]. In c9FTD/ALS, neuronal inclusions of c9RAN proteins are similarly present in vulnerable areas (e.g., neurons of neocortex and hippocampus), but there is a paucity of such inclusions in certain affected areas, such as the spinal cord [3, 25]. As with other aggregation-prone proteins involved in neurodegeneration (e.g., tau [14]), it remains unclear whether c9RAN inclusions per se are toxic, or whether RAN translation of transcripts from the *C9ORF72* repeat contributes to neurodegeneration through the formation of toxic, soluble oligomers.

As with RAN translation, the consequence of foci formation in c9FTD/ALS is currently under investigation. Taking cues from other repeat expansion disorders, it is anticipated that foci will sequester select RNA-binding proteins, and cause the misregulation of crucial downstream RNA targets. To date, studies have identified several proteins that bind GGGGCC transcripts [24, 29, 38]. The findings herein highlight the necessity to similarly identify proteins bound and sequestered by CCCCGG foci.

Examination of foci formed of sense or antisense transcripts of the expanded *C9ORF72* repeat revealed that, in addition to the frontal cortex and spinal cord, RNA foci accumulate in the cerebellum. Together with other studies, it is now evident that numerous pathological features are present in the cerebellum of c9FTD/ALS patients, including nuclear RNA foci, c9RAN protein inclusions, as well as p62-positive inclusions [1, 3, 6, 25, 26]. Furthermore, FTD cases caused by the *C9ORF72* repeat expansion show atrophy of the parietal lobe and cerebellum, in addition to frontal and temporal lobe atrophy [37]. In fact, c9FTD is characterized by greater cerebellar atrophy than sporadic FTD, as well as FTD caused by mutations in *MAPT* [37]. Consequently, cerebellar atrophy, nuclear RNA foci, and proteinaceous inclusions may be considered characteristic features of c9FTD/ALS. Future studies of c9FTD/ALS should therefore encompass evaluations of the cerebellum which, to date, has been largely neglected.

Of interest, foci and poly(GP) inclusions were seldom observed in the same cell in the frontal cortex and cerebellum of c9FTD/ALS patients. Although it is possible that foci too small to be easily detected are present in cells with poly(GP) inclusions, our findings suggest that only those (GGGGCC)_exp_ or (CCCCGG)_exp_ transcripts that escape being sequestered as foci, and are instead exported to the cytoplasm, become available for RAN translation. That RNA foci are found in both neuronal and glial cells (Figs. 4, 5), while poly(GP) inclusions are neuronal [3], coupled with the fact that RNA foci in the cerebellum are found in greatest abundance in cells in proximity to the Purkinje cell layer, whereas poly(GP) inclusions are widely expressed throughout the molecular and granule layers, does provide a basis for the lack of co-occurrence of these two features in a given cell. While it remains to be determined whether other c9RAN proteins are more frequently found in the same cells as foci, and whether both sense and antisense foci are present within the same cell, these findings support the notion that foci and inclusions represent two distinct pathogenic mechanisms for *C9ORF72* repeat expansions.

Since the 2011 discovery that the expanded hexanucleotide repeat in *C9ORF72* causes chromosome 9p-linked FTD and ALS [11, 30], several neuropathological features unique to c9FTD/ALS have been identified [1, 3, 5, 7, 11, 24, 25, 28]. The findings from the present study expand this list and highlight the need to broaden our view of potential disease mechanisms to include toxicity potential stemming from the antisense transcript. Going forward, it will be critical to distinguish if and how RNA transcripts and c9RAN proteins contribute to the pathogenesis of disease, and whether the frequency or regional localization of RNA foci and c9RAN inclusions correlate with distinct clinical features. While these questions are being investigated, c9RAN proteins should be explored as a biomarker for c9FTD/ALS, as should treatment strategies aimed at eradicating the putatively toxic sense and antisense transcripts responsible for both foci formation and RAN translation.

## Electronic supplementary material

Below is the link to the electronic supplementary material.

Supplementary material 1 (DOCX 837 kb)
