# Supplementary material for: Antisense transcripts of the expanded C9ORF72 hexanucleotide repeat form nuclear RNA foci and undergo repeat-associated non-ATG translation in c9FTD/ALS
Source: Acta Neuropathol. 2013 Oct 16;126(6):829–44. doi: 10.1007/s00401-013-1192-8 (PMC3830741; doi:10.1007/s00401-013-1192-8)

**Acta Neuropathologica**

**Electronic Supplementary Material**

**Antisense transcripts of the expanded *C9ORF72* hexanucleotide repeat form nuclear RNA foci and undergo repeat-associated non-ATG translation in c9FTD/ALS**

Tania F. Gendron^1#^, Kevin F. Bieniek^1,2#^, Yong-Jie Zhang^1^, Karen Jansen-West^1^, Peter E.A. Ash^3^, Thomas Caulfield^1^, Lillian Daughrity^1^, Judith H. Dunmore^1^, Jeannie Chew^1,2^, Monica Castanedes-Casey^1^, Danielle M. Cosio^1^, Marka van Blitterswijk^1^, Wing C. Lee^1^, Rosa Rademakers^1^, Kevin B. Boylan^4^, Dennis W. Dickson^1^* and Leonard Petrucelli^1^*

^1^ Department of Neuroscience, Mayo Clinic Florida, Jacksonville, FL 32224, USA.

^2^ Mayo Graduate School, Mayo Clinic College of Medicine, Rochester, MN 55905, USA.

^3^ Department of Pharmacology, Boston University School of Medicine, Boston, MA 02118, USA.

^4^ Department of Neurology, Mayo Clinic Florida, Jacksonville, FL 32224, USA.

^#^ These authors contributed equally to this work.

***** Corresponding Authors:

Leonard Petrucelli, PhD

Phone: 904-953-2855

Fax: 904-953-6276

Email: [petrucelli.leonard@mayo.edu](mailto:petrucelli.leonard@mayo.edu)

Dennis W. Dickson, MD

Phone: 904-953-7137

Fax: 904-953-7117

Email: dickson.dennis@mayo.edu


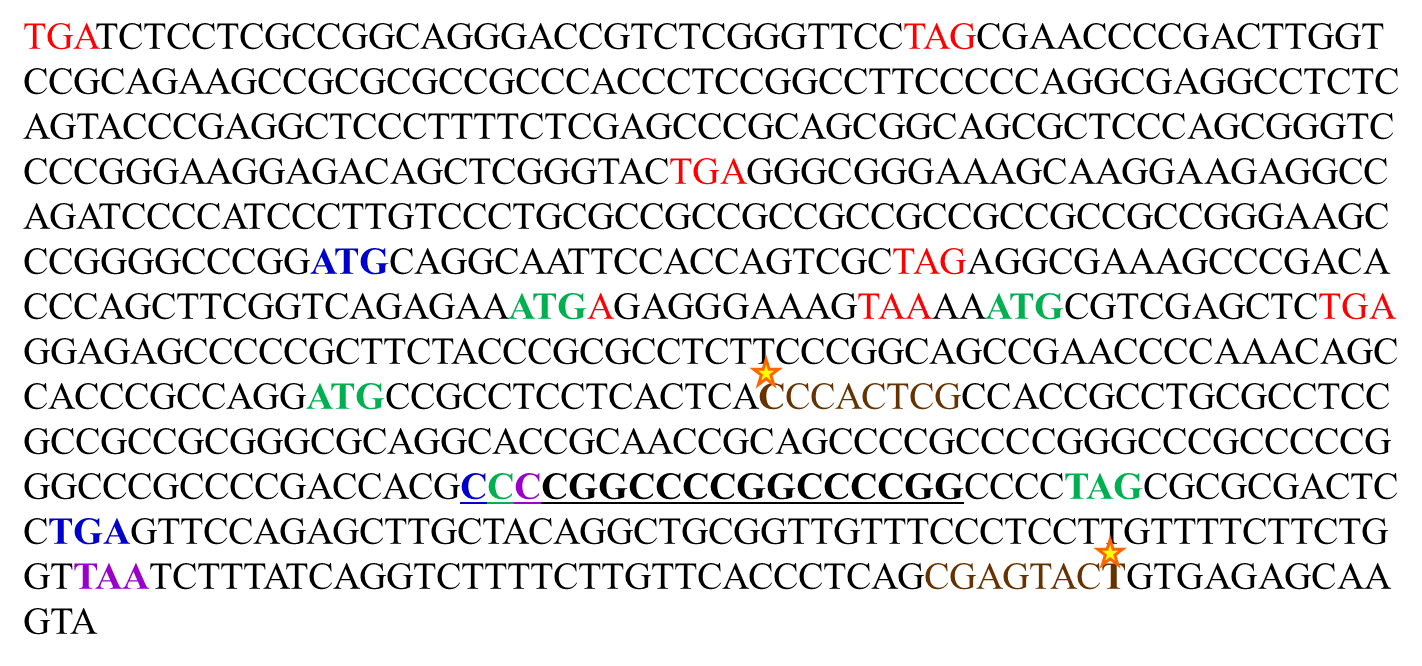


**Online Resource 1. Genomic sequence of the antisense strand of the *C9ORF72* repeat region.** Shown are 552 bp upstream of the first CCCCGG (bold/underline) repeat sequence and 130 bp downstream of the last CCCCGG repeat sequence. Note that the first 3 C’s of the repeat are assigned a different color (blue, green, purple) to distinguish the first bp in the 3 alternate reading frames, respectively. CCCCGG initiates poly(PR) translation. One ATG start codon begins 273 bp upstream of the first CCCCGG repeat, and a TGA stop codon begins 19 bp downstream of the last CCCCGG repeat; these are indicated in blue bolded text. CCCGGC initiates poly(PG) translation. Three ATG start codons begin 114, 195 and 213 bp upstream of the first CCCGGC repeat, and a TGA stop codon begins 4 bp downstream of the last CCCGGC repeat; these are indicated in green bolded text. CCGGCC initiates poly(PA) translation. No ATG start codon is upstream of the first CCGGCC repeat but a TAA stop codon begins 73 bp downstream of the last CCGGCC repeat (indicated in purple bolded text). Additional ATG-initiated translation sites not indicated here are found further upstream the repeat. However, stop codons (indicated in red) would stop translation from these sites before the repeat is translated.


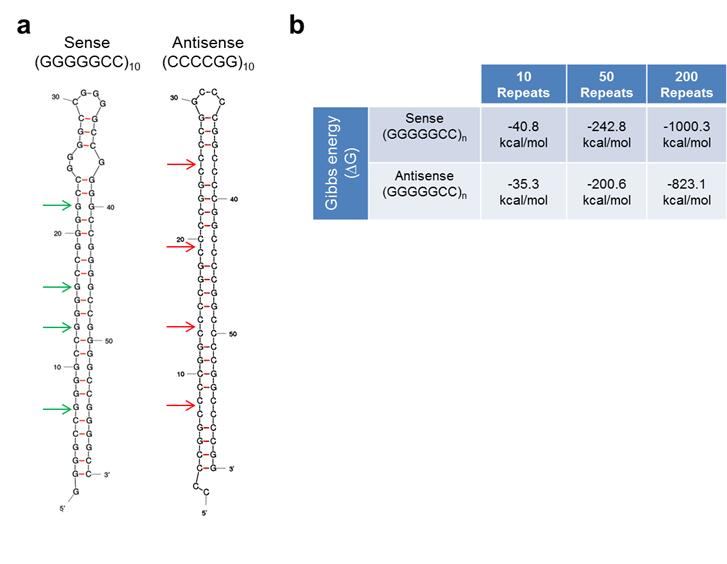


**Online Resource 2.** **Secondary structure prediction for the CCCCGG repeat sequence.** Predicted secondary structures for 10 repeats of GGGGCC or CCCCGG (**a**), and Gibbs energy (**b**). Red arrows on the antisense strand indicate sets of four unpaired C nucleotides within the stem, whereas green arrows on the sense strand indicate unpaired GG bases.

**Online Resource 3. Additional antibody characterization for c9RAN proteins.** (**a**) The immunoreactivity of antibodies to c9RAN proteins towards (GA)_8_ and (GR)_8_ peptides was measured by adsorbing peptides onto carbon electrodes in 96-well MSD plates, and co-incubating wells with anti-PA, anti-PR, anti-GP, anti-GA or anti-GR antibodies, and a SULFO-tagged anti-rabbit secondary antibody. Antibody binding to respective peptides was quantified by measuring the intensity of emitted light upon electrochemical stimulation of the plate using the MSD Sector Imager 2400. For each pair of antibodies, binding responses were normalized to the signal of the antibody showing the highest binding to its respective antigen. Error bars indicate standard deviations from duplicate wells. (**b**) Western blot analysis of lysates from HEK293T cells transfected to express enhanced GFP-tagged (PA)_5_, (PR)_5,_ (GP)_5_, (GA)_5,_ or (GR)_5_. Blots were probed with the indicated antibodies. (**c**) Immunofluorescence staining of HEK293T cells transfected to express the enhanced GFP (green)-tagged (GA)_5_ or (GR)_5_ with the indicated antibodies (red). Nuclei are stained with Hoechst (blue). Scale bar = 10 µM.


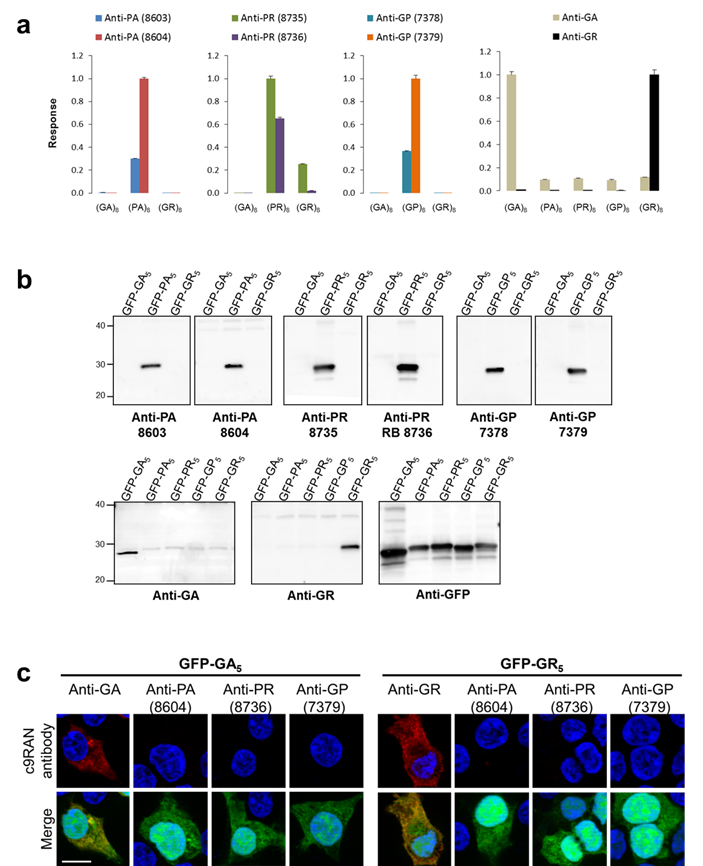

Supplement: Supplementary file 1 — Supplementary material 1 (DOCX 837 kb) [file 401_2013_1192_MOESM1_ESM.docx]
